# Prognostic Significance of Anti-Aminoacyl-tRNA Synthetase Antibodies in Polymyositis/Dermatomyositis-Associated Interstitial Lung Disease: A Retrospective Case Control Study

**DOI:** 10.1371/journal.pone.0120313

**Published:** 2015-03-19

**Authors:** Hironao Hozumi, Noriyuki Enomoto, Masato Kono, Tomoyuki Fujisawa, Naoki Inui, Yutaro Nakamura, Hiromitsu Sumikawa, Takeshi Johkoh, Ran Nakashima, Yoshitaka Imura, Tsuneyo Mimori, Takafumi Suda

**Affiliations:** 1 Second Division, Department of Internal Medicine, Hamamatsu University School of Medicine, Hamamatsu, Shizuoka, Japan; 2 Department of Clinical Pharmacology and Therapeutics, Hamamatsu University School of Medicine, Hamamatsu, Shizuoka, Japan; 3 Diagnostic and Interventional Radiology, Osaka University Graduate School of Medicine, Suita, Osaka, Japan; 4 Department of Radiology, Kinki Central Hospital of Mutual Aid Association of Public School Teachers, Itami-city, Hyogo, Japan; 5 Department of Rheumatology and Clinical Immunology, Graduate School of Medicine, Kyoto University, Kyoto, Japan; Medical University of South Carolina, UNITED STATES

## Abstract

**Background:**

In polymyositis/dermatomyositis (PM/DM), anti-aminoacyl-tRNA synthetase (ARS) antibodies are closely associated with interstitial lung disease (ILD), a frequent pulmonary complication. However, the clinical significance of anti-ARS antibodies is not well established.

**Objective:**

We aimed to evaluate the clinical significance of anti-ARS antibodies in PM/DM-ILD patients.

**Methods:**

Forty-eight consecutive PM/DM-ILD patients were studied retrospectively. Anti-ARS antibodies were screened by ELISA and confirmed by RNA immunoprecipitation test. Medical records, high-resolution computed tomography images, and surgical lung biopsy specimens were compared between ARS-positive (ARS group) and ARS-negative patients (non-ARS group).

**Results:**

Anti-ARS antibodies were detected in 23 of 48 patients (48%). Radiologically, nonspecific interstitial pneumonia (NSIP) pattern was observed more frequently in the ARS group than in the non-ARS group (73.9% vs. 40%, *P* = 0.02). Pathologically, NSIP was the most frequent in both groups. Ten-year survival rate was also significantly higher in the ARS group than in the non-ARS group (91.6% vs. 58.7%, *P* = 0.02). Univariate Cox hazards analysis revealed that the presence of anti-ARS antibodies was associated with better prognosis (HR = 0.34, 95% CI 0.08–0.80; *P* = 0.01).

**Conclusions:**

The presence of anti-ARS antibodies is a possible prognostic marker in patients with PM/DM-ILD.

## Introduction

Idiopathic inflammatory myopathy (IIM) comprises a group of systemic autoimmune disorders, including polymyositis (PM) and dermatomyositis (DM), affecting skeletal muscles and other organs [1–3]. In patients with PM/DM, interstitial lung disease (ILD) is a common extramuscular involvement associated with poor prognosis [4–6]. We previously described the clinical features of ILD-associated PM/DM (PM/DM-ILD) [7, 8] and identified the prognostic factors based on the clinical characteristics of a large series of PM/DM-ILD patients [9].

Accumulating evidence supports an association between ILD and the presence of certain myositis-specific autoantibodies (MSAs); in particular, anti-aminoacyl tRNA-synthetase enzyme (ARS) antibodies and anti-melanoma differentiation-associated gene 5 (MDA-5) antibody (also termed anti-CADM-140 antibody) are more closely associated with ILD than other MSAs [10–15]. Anti-ARS antibodies were detected in approximately 50% of PM/DM-ILD patients [11]. To date, eight types of anti-ARS antibodies (Jo-1, PL-7, PL-12, EJ, OJ, KS, Zo, and Ha) have been identified [10, 16]. Although patients with different types of anti-ARS antibodies show some unique clinical features and prognosis [17–21], these patient subgroups can also present with similar clinical manifestations, such as ILD, myositis, arthritis, Raynaud’s phenomenon, and “mechanic’s hands” [also known as anti-synthetase syndrome (ASS)] [16, 17].

Yoshifuji *et al*. reported that the response to initial therapy is better in anti-ARS-positive PM/DM-ILD than in anti-ARS-negative PM/DM-ILD [11]. However, there have been no studies comparing the radiological and pathological features between anti-ARS-negative and anti-ARS-positive PM/DM-ILD patients or assessing the long-term prognostic significance of anti-ARS antibodies.

In contrast, several studies have demonstrated the clinical significance of anti-MDA-5 antibody, which is exclusively detected in DM or clinically amyopathic DM (CADM) [13–15, 17]. Patients positive for anti-MDA-5 antibody more often developed rapidly progressive and fatal ILD [13–15] compared with those positive for anti-ARS antibodies [15]. Anti-MDA-5 and anti-ARS antibodies are mutually exclusive [17]. Therefore, the early discrimination between patients positive for anti-ARS antibodies and anti-MDA-5 antibody is crucial for determining the appropriate treatment strategy for PM/DM-ILD.

At present, anti-MDA-5 antibody can be measured only in specialized facilities by protein immunoprecipitation tests or enzyme-linked immunosorbent assay (ELISA) [14, 17, 22]. Conversely, anti-ARS antibodies are measurable using a commercially available ELISA kit (MESACUP anti-ARS test, MBL, Nagoya, Japan) or a line blot assay kit (EUROLINE Myositis Profile 3, EUROIMMUN AG, Luebeck, Germany) [23]. We previously demonstrated that this ELISA kit can detect anti-ARS antibodies with sensitivity and specificity comparable to the RNA immunoprecipitation (RNA-IP) test [23]. Here using this ELISA kit, we aimed to describe the clinical, radiological, and pathological features of PM/DM-ILD and compare the prognosis of anti-ARS-positive to anti-ARS-negative PM/DM-ILD patients.

## Materials and Methods

### Subjects

The institutional review board of Hamamatsu University School of Medicine approved this study (approval number 25–225) and waived patient approval or informed consent because the study involved a retrospective review of patient records, images, and pathological specimens. This information was notified on the website (http://hamamatsu-lung.com/study.html).

We retrospectively reviewed consecutive patients diagnosed with PM/DM-ILD between 1995 and 2013 at Hamamatsu University Hospital (Hamamatsu, Japan). Of the 56 patients identified, 8 were excluded because of overlapping connective tissue diseases (CTDs) (5 patients with Sjögren’s syndrome, 1 with systemic sclerosis, 1 with rheumatoid arthritis, and 1 with systemic lupus erythematosus). There were no patients who had active malignancies at initial diagnosis. Finally, 48 PM/DM-ILD patients were included in this study.

The diagnosis of PM/DM was confirmed on the basis of Bohan and Peter’s criteria [1, 2]. In this study, patients with definite or probable PM/DM were included. CADM was diagnosed as a distinct subgroup of DM when the patient had a skin rash characteristic of DM without the clinical evidence of muscle disease and with little or no increase in serum creatine kinase (CK) during the study period [3, 7, 9, 14, 15, 24].

ILD was diagnosed based on clinical presentation, pulmonary function tests, high-resolution computed tomography (HRCT) images, and lung biopsy findings [25–28]. All patients underwent transbronchial lung biopsy and/or bronchoalveolar lavage (BAL), and 27 patients (56%) also underwent surgical lung biopsy (SLB). Patients with other known causes of ILD were excluded [25–28].

Disease onset type was classified as ILD-preceding type if ILD diagnosis preceded PM/DM diagnosis by three months or longer, concomitant onset type if ILD and PM/DM were diagnosed within 3 months, or myositis-preceding type if PM/DM diagnosis preceded ILD diagnosis by 3 months or longer [4, 7]. ILD form was classified as acute/subacute (lasting less than 3 months from the onset) or chronic (lasting 3 months or longer) according to the clinical presentation [7, 9]. For the assessment of clinical course, improvement and deterioration of ILD were defined based on the International Consensus Statement of idiopathic pulmonary fibrosis (IPF) [28] with slight modification by the consensus of 2 lung physicians specializing in ILD. Briefly, improvement or deterioration were defined by two or more of the following: (1) a decrease or increase in respiratory symptom severity, (2) a decrease or increase in parenchymal abnormality on chest HRCT scan, and/or (3) a ≥10% increase or decrease in percent predicted forced vital capacity (%FVC) or a ≥10 Torr increase or decrease in arterial oxygen pressure (PaO_2_).

### Detection of anti-ARS antibodies

For all 48 PM/DM-ILD patients diagnosed at Hamamatsu University Hospital, the serum samples stored since the initial ILD diagnosis were available. The samples were screened using a recently developed ELISA kit (MESACUP anti-ARS test, MBL, Nagoya, Japan) that can detect five anti-ARS antibodies (Jo-1, PL-7, PL-12, EJ, and KS) [23]. If positive, the presence of anti-ARS antibodies was confirmed by RNA-IP [23].

**Fig 1:**
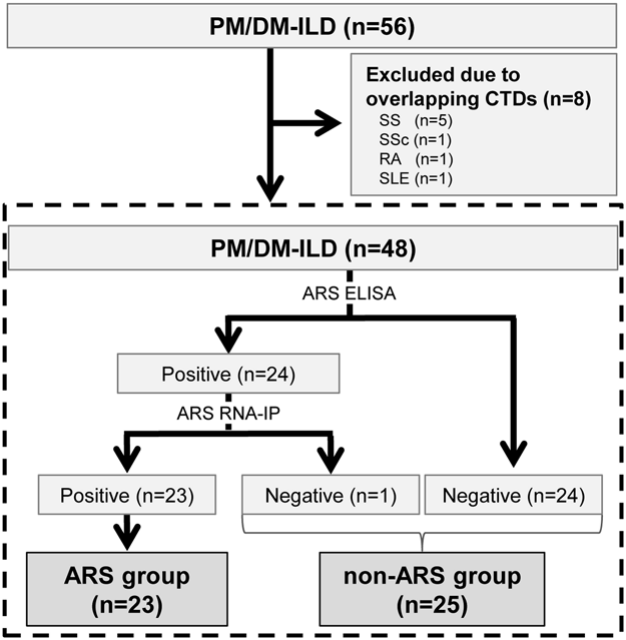
Number of patients included in this study and disease classification. Of 56 patients identified, 8 patients were excluded because of comorbid connective tissue diseases (CTDs) (5 patients with Sjögren’s syndrome, 1 with systemic sclerosis, 1 with rheumatoid arthritis, and 1 with systemic lupus erythematosus). There were no patients who had active malignancies at initial diagnosis. Finally, 48 PM/DM-ILD patients were included in this study. PM, polymyositis; DM, dermatomyositis; ILD, interstitial lung disease; CTD, connective tissue disease; SS, Sjögren syndrome; SSc, systemic sclerosis; RA, rheumatoid arthritis; SLE, systemic lupus erythematosus; ELISA, enzyme-linked immunosorbent assay; RNA-IP, RNA immunoprecipitation.

Of the 48 PM/DM-ILD patients meeting the inclusion criteria, anti-ARS antibodies were detected in 24 using the ELISA kit, of which 23 were also anti-ARS-positive by RNA-IP (termed ARS group). The one patient positive for anti-ARS antibody by ELISA but negative by RNA-IP, and the 24 patients negative for anti-ARS antibodies by ELISA were classified as negative; thus, 25 patients were classified as negative for anti-ARS antibodies (non-ARS group) (Fig. 1). Anti-ARS antibodies detected in this study included PL-7 in 8 patients, Jo-1 in 6, PL-12 in 4, KS in 2, EJ in 2, and KS + EJ in 1.

### Review of radiographical findings

HRCT images acquired at initial ILD diagnosis were reviewed. These images comprised 1–2.5-mm collimation sections at 10-mm intervals. They were reconstructed by a high spatial frequency algorithm and displayed at window settings appropriate for viewing the lung parenchyma (window level, -600 to -800 Hounsfield units; window width, 1200 to 2000 Hounsfield units). Images were randomized and reviewed independently by two expert chest radiologists unaware of the related clinical information. The images were assessed for the presence and distribution of lung parenchymal abnormalities on HRCT findings.

The predominant distribution of abnormalities on HRCT findings was evaluated according to the following criteria. Cranio-caudal predominance was assessed as “upper” when most of the abnormal HRCT findings were above the level of the tracheal carina, as “lower” when most of the abnormalities were below the level of the tracheal carina, and as “random/diffuse” when most of the abnormalities were distributed randomly or diffusely. The axial distribution was classified as “peripheral” if the abnormalities were present primarily in the outer third of the lung, “peribronchovascular” if abnormalities were primarily around the bronchus and artery, and “diffuse” if abnormalities were distributed diffusely.

HRCT findings, including ground-glass opacity (GGO), consolidation, reticular opacity, honeycombing, traction bronchiectasis, non-septal linear opacity/subpleural curvilinear line (SCLL), emphysema, and lower lobe volume loss were interpreted according to Fleischner’s criteria with slight modification [29] (S1 Protocol). Each CT finding was recorded as present or absent.

HRCT pattern was classified as usual interstitial pneumonia (UIP) pattern, possible UIP pattern, or inconsistent with UIP pattern according to the guidelines for IPF with slight modification [26]. The cases interpreted as inconsistent with UIP pattern were further classified as nonspecific interstitial pneumonia (NSIP) pattern or organizing pneumonia (OP) pattern according to the guidelines for idiopathic interstitial pneumonias (IIPs) [25, 27]. Patterns that could not be classified as NSIP or OP were categorized as unclassifiable pattern (S2 Protocol). Disagreements regarding HRCT interpretation were resolved by consensus between the two radiologists.

### Review of pathological findings

Surgical lung biopsy specimens obtained from at least two sites in each patient were reviewed. The pathological classification (UIP, NSIP, or OP) was based on the guidelines for IPF and IIPs [25–27]. Pathological patterns that could not be classified according to these criteria were categorized collectively as an unclassifiable interstitial pneumonia pattern [27].

Each of the following pathological findings was scored semiquantitatively (absent, 0; mild, 1; moderate, 2; and severe, 3): fibroblastic foci, alveolar wall fibrosis, alveolar wall inflammation, intra-alveolar cellularity, and organization. The presence or absence of the following findings was also evaluated: microscopic honeycombing, prominent plasmacytes, dense perivascular collagen, lymphoid aggregate with germinal center, and extensive pleuritis [30, 31].

Pathological patterns and the scoring of pathological findings were evaluated independently by two lung pathologists and the final diagnoses were made by consensus.

### Statistical analysis

All values are expressed as median (range) or number (%). Depending on the sample size, either the Fisher’s test or chi-square test was used for comparing proportions among groups. The Mann—Whitney U test was used for comparing medians. Interobserver agreement on HRCT diagnosis was analyzed using the κ statistic test and classified as follows: poor (κ = 0–0.20), fair (κ = 0.21–0.40), moderate (κ = 0.41–0.60), good (κ = 0.61–0.80), and excellent (κ = 0.81–1.00). The observation period for survival was calculated from the date of initial diagnosis of ILD (not PM/DM diagnosis) to the last visit or the time of death. Survival was evaluated using the Kaplan—Meier method and survival curves were compared by the log-rank test. Cox hazards analysis was used to identify variables associated with survival. In all analyses, *P* < 0.05 was considered statistically significant. All data were analyzed using commercially available software (JMP version 9.0.3a, SAS Institute Inc, Cary, NC, USA).

## Results

### Clinical characteristics

The clinical characteristics of the ARS and non-ARS groups are summarized in Table 1. The proportion of females was significantly higher in the ARS group than in the non-ARS group (82.6% vs. 48.0%, *P* = 0.017). There were no statistically significant group differences in age at ILD or PM/DM diagnosis, smoking status, disease onset type, ILD form, IIM type, or observation period.

**Table 1:** Patient characteristics.

	ARS, n = 23	non-ARS, n = 25	*P* value
**Median age, yeas (range)**			
at ILD diagnosis	55 (37–76)	55 (32–75)	0.66
at PM/DM diagnosis	54 (38–76)	55 (32–75)	0.56
**Females, n (%)**	19 (82.6)	12 (48.0)	0.017*
**Smoking status, n (%)**			0.21
Never	15 (65.2)	14 (56.0)	
Former	2 (8.7)	7 (28.0)	
Current	6 (26.1)	4 (16.0)	
**Disease onset type**			0.17
ILD-preceding	5 (21.7)	2 (8.0)	
Concomitant onset	15 (65.2)	22 (88.0)	
PM/DM-preceding	3 (13.0)	1 (4.0)	
**ILD form**			0.15
Acute/subacute	7 (30.4)	13 (52.0)	
Chronic	16 (69.6)	12 (48.0)	
**IIM type**			0.18
PM	1 (4.3)	5 (20.0)	
DM	8 (34.8)	10 (40.0)	
CADM	14 (60.9)	10 (40.0)	
**Median observation period, years (range)**	5.7 (1.1–12.7)	3.6 (0.2–19.2)	0.16

Data are presented as n (%), median (range).

*P < 0.05

ILD, interstitial lung disease; IIM, Idiopathic inflammatory myopathy; PM, polymyositis; DM, dermatomyositis; CADM, clinically amyopathic dermatomyositis.

### Clinical symptoms, laboratory findings, pulmonary function test results, and BAL findings

The clinical symptoms, laboratory findings, pulmonary function test results, and BAL findings at ILD diagnosis are presented in Table 2. Muscle weakness/myalgia was more frequently observed in the non-ARS group than in the ARS group (52.4% vs. 17.4%, *P* = 0.02). Median CK and aldolase levels were significantly higher in the non-ARS group than the ARS group (*P* = 0.017 and *P* = 0.013, respectively). Median PaO_2_ level was significantly lower in the non-ARS group than in the ARS group (*P* = 0.04). Percent predicted forced vital capacity (%FVC) was moderately low in both groups with no significant group difference.

**Table 2:** Clinical symptoms, laboratory findings, pulmonary function test results, and bronchoalveolar lavage findings at ILD diagnosis.

	ARS, n = 23	non-ARS, n = 25	*P* value
**Clinical symptom, n (%)**			
Cough	15 (65.2)	13 (52.0)	0.39
Dyspnea on exertion	11 (47.8)	14 (56.0)	0.77
Muscle weakness/Myalgia	4 (17.4)	13 (52.0)	0.02*
**Laboratory findings, median (range)**			
CK, IU/L	87 (30–798)	281 (24–5274)	0.017*
Aldolase, IU/L	5.6 (3.1–19.1)	11 (3.3–133)	0.018*
KL-6, U/mL	962 (422–3250)	759 (254–2450)	0.81
PaO_2_, Torr	80 (63–105)	72 (47.9–103)	0.04*
**Pulmonary function tests, median (range)**			
FVC, % predicted	66.5 (42.5–93.0)	65.9 (40.6–107.7)	0.99
FEV_1.0_/FVC, %	83.1 (68.1–73.7)	85.4 (73.7–105)	0.22
**BAL findings, median (range)**			
Lymphocytes, %	10.7 (1.2–70.0)	6.4 (0.6–32.0)	0.26
Neutrophils, %	0.7 (0–14.0)	0.6 (0–31.6)	0.33
Eosinophils, %	2.0 (0–10.2)	0.8 (0–18.6)	0.08
CD4/8 ratio	0.51 (0.07–4.97)	0.66 (0.05–3.82)	0.85

Data are presented as n (%), median (range).

*P < 0.05

CK, creatine kinase; PaO_2_, arterial oxygen pressure; FVC, forced vital capacity; FEV_1.0_, forced expiratory volume 1.0(sec); BAL, bronchoalveolar lavage.

### HRCT distributions, findings, and patterns

Chest HRCT images at ILD diagnosis were available for all patients (Table 3). In both the ARS and non-ARS groups, abnormal HRCT findings were predominantly distributed in the lower lung zone and peripheral and/or peribronchovascular region. GGO, traction bronchiectasis, and lower lobe volume loss were frequently observed in both groups, whereas little or no honeycombing was seen in either group. There were no statistically significant differences in the frequencies of specific findings or distributions between groups. HRCT pattern in all patients was inconsistent with UIP pattern. The NSIP pattern was found in 17 ARS group patients (73.9%) but only in 10 non-ARS group patients (40%). Conversely, the unclassifiable pattern was observed in only 6 ARS group patients (26.1%) but in 11 non-ARS group patients (44%). There was a significant difference in pattern between the two groups (*P* = 0.02).

**Table 3:** HRCT distributions, findings, and patterns.

	ARS, n = 23	non-ARS, n = 25	*P* value
**Cranio-caudal distribution**			0.23
Upper predominance	0 (0)	0 (0)	
Lower predominance	23 (100)	22 (88.0)	
Random/diffuse	0 (0)	3 (12.0)	
**Axial distribution**			
Peripheral	14 (60.1)	20 (80.0)	0.21
Peribronchovascular	14 (60.1)	15 (60.0)	1.00
Diffuse	5 (21.7)	4 (16.0)	0.72
**HRCT findings**			
Ground-glass opacity	23 (100)	22 (88.0)	0.24
Consolidation	13 (56.5)	14 (56)	1.00
Reticular opacities	9 (39.1)	10 (40.0)	1.00
Honeycombing	0 (0)	1 (4)	1.00
Traction bronchiectasis	21 (91.3)	19 (76.0)	0.25
Nonseptal linear opacities/SCLL	17 (73.9)	15 (60.0)	0.37
Emphysema	1 (4.0)	5 (20.0)	0.19
Lower volume loss	22 (95.7)	21 (84.0)	0.35
**HRCT patterns**			0.03*
UIP/possible UIP	0 (0)	0 (0)	
NSIP	17 (73.9)	10 (40)	
OP	0 (0)	4 (16)	
Unclassifiable	6 (26.1)	11 (44)	

Data are presented as n (%), median (range).

Interobserver agreement on HRCT distributions, findings, and patterns between both radiologists was fair to good (κ = 0.37–0.79).

*P < 0.05

HRCT, high-resolution computed tomography; SCLL, subpleural curve linear line; UIP, usual interstitial pneumonia; NSIP, nonspecific interstitial pneumonia; OP, organizing pneumonia.

### Pathological patterns and findings

Of the 48 patients, 27 underwent SLB. Pathological patterns and findings are shown in Table 4. The pathological patterns of the ARS group patients with available SLB findings (n = 13) included NSIP in 12 (92%) and UIP in 1 (8%), whereas those of the non-ARS group (n = 14) included NSIP in 11 patients (79%), UIP in 2 (14%), and unclassifiable interstitial pneumonia in 1 (7%). There was no statistically significant difference in pathological pattern frequency distribution between the two groups (*P* = 0.51). There were no significant differences in the frequencies of various pathological findings between ARS and non-ARS groups, including fibroblastic foci [4 (30.8%) vs. 2 (14.3%)], microscopic honeycombing [5 (38.5%) vs. 4 (28.6%)], prominent plasmacytes [7 (53.8%) vs. 8 (57.1%)], and lymphoid aggregate with germinal center [7 (53.9%) vs. 9 (64.3%)], although organization [6 (46.2%) vs. 10 (71.4%)] tended to be less frequent in ARS group.

**Table 4:** Pathological patterns and findings.

	ARS, n = 13	non-ARS, n = 14	*P* value
**Patterns, n (%)**			0.51
NSIP	12 (92)	11 (79)	
UIP	1 (8)	2 (14)	
Unclassifiable	0 (0)	1 (7)	
**Score, none/mild/moderate/severe**			
Fibroblastic foci	9/4/0/0	12/1/1/0	0.20
Alveolar wall fibrosis	0/5/3/5	0/7/4/3	0.62
Alveolar wall inflammation	0/1/12/0	0/4/10/0	0.33
Intra-alveolar cellularity	0/13/0/0	0/11/3/0	0.22
Organization	7/3/3/0	4/4/6/0	0.38
**Prevalence, n (%)**			
Microscopic honeycombing	5 (38.5)	4 (28.6)	0.69
Prominent plasmacytes	7 (53.8)	8 (57.1)	0.86
Dense perivascular collagen	1 (7.7)	1 (7.1)	0.95
Lymphoid aggregate with germinal center	7 (53.9)	9 (64.3)	0.70
Extensive pleuritis	1 (7.7)	1 (7.1)	0.95

Data are presented as n (%).

NSIP, nonspecific interstitial pneumonia; UIP, usual interstitial pneumonia.

### Treatment and outcome

**Fig 2:**
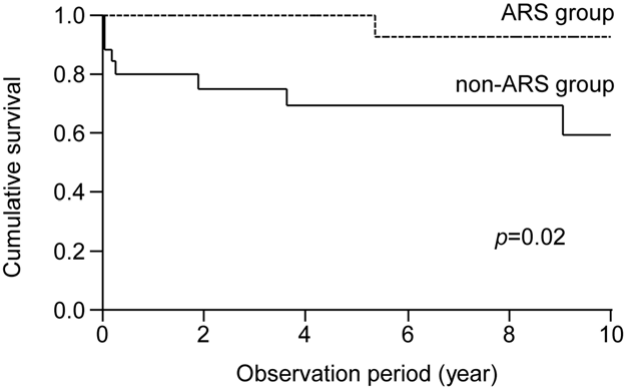
Kaplan—Meier survival curves. The 5-year and 10-year survival rates were higher in the ARS group than in the non-ARS group (5-year: 100% vs. 69.1%; 10-year: 92.3% vs. 40.8%, P = 0.02 by log-rank test).

Twenty-two of 23 patients in the ARS group (95.7%) and 23 of 25 in the non-ARS group (92.0%) were treated for PM/DM-ILD during the observation period (Table 5). Although there was no statistically significant difference in the treatment regimen between the two groups (*P* = 0.22), ARS group patients exhibited a significantly higher response rate (100% vs. 78.3%, *P* = 0.049). In the ARS group, only 1 of 23 patients died (due to cancer of unknown primary origin) during the observation period (4.4%), whereas 8 of 25 non-ARS patients (32.0%) died during the observation period (6 from respiratory failure, 1 from oropharyngeal cancer, and 1 from rupture of an abdominal aortic aneurism). Both the overall death rate and the death rate from respiratory failure were significantly lower in the ARS group (*P* = 0.02 and *P* = 0.02, respectively). Kaplan—Meier survival curves are shown in Fig. 2. The 5-year and 10-year survival rates were higher in the ARS group than in the non-ARS group (5-year: 100% vs. 69.1%; 10-year: 92.3% vs. 40.8%, P = 0.02 by log-rank test).

**Table 5:** Treatment and outcome.

	ARS, n = 23	non-ARS, n = 25	*P* value
**Treatment, yes, n (%)**	22 (95.7)	23 (92.0)	0.86
**Treatment regimen, n (%)**			0.22
Prednisolone alone	10 (45.5)	6 (26.1)	
Prednisolone + Cyclosporin	11 (50.0)	14 (60.9)	
Prednisolone + Cyclophosphamide	1 (4.5)	1 (4.3)	
Prednisolone + Tacrolimus	0 (0)	2 (8.7)	
**Improvement by initial treatment, yes, n(%)**	22 (100)	18 (78.3)	0.049*
**Death during observation period, n (%)**	1 (4.4)	8 (32.0)	0.02*
Due to respiratory failure, n (%)	0 (0)	6 (24.0)	0.02*

Data are presented as n (%), median (range).

*P < 0.05

Univariate analysis revealed that seropositive status for anti-ARS antibodies (HR = 0.34; 95% CI 0.08–0.80; *P* = 0.01) was a favorable prognostic factor. In addition, higher PaO_2_ levels at initial ILD diagnosis (HR = 0.93, 95% CI 0.89–0.98; *P* = 0.007) were associated with longer survival, whereas the acute/subacute ILD form was related to poorer prognosis (HR = 3.70, 95% CI 1.59–16.7; *P* = 0.001) (Table 6). There were no significant differences in treatment response and outcome among patients exhibiting each anti-ARS antibody detected by RNA-IP (data not shown).

**Table 6:** Univariate Cox hazards analysis for survival.

	HR	95%CI	*P* value
Anti-ARS-antibodies, positive	0.34	0.08–0.80	0.01*
Sex, female	0.82	0.42–1.65	0.55
Age at ILD diagnosis, years	1.01	0.94–1.08	0.73
Age at PM/DM diagnosis, years	1.01	0.94–1.08	0.80
Never smoked, yes	0.88	0.45–1.78	0.71
ILD form, acute/subacute	3.70	1.59–16.7	0.001*
PaO_2_ at initial ILD diagnosis, torr	0.93	0.89–0.98	0.007*
%FVC at initial ILD diagnosis, %	0.95	0.90–1.002	0.06

*P < 0.05

HR, hazard ratio; 95%CI, 95% confidence interval; ILD, interstitial lung disease; PM, polymyositis; DM, dermatomyositis; CADM, clinically amyopathic dermatomyositis; PaO_2_, arterial oxygen pressure; %FVC, predicted forced vital capacity.

## Discussion

The present study was conducted to elucidate the clinical significance of anti-ARS antibodies in PM/DM-ILD. We found that 48% of PM/DM-ILD patients were positive for anti-ARS antibodies and that these patients showed a female predominance and less frequent myositis compared with non-ARS patients. Radiologically, NSIP pattern was more frequently observed in the ARS group than in the non-ARS group. Furthermore, the presence of anti-ARS antibodies was associated with favorable treatment response and greater survival. To the best of our knowledge, this is the first study to compare the clinical, radiological, and pathological features and clinical outcomes between anti-ARS-positive and anti-ARS-negative PM/DM-ILD patients, providing valuable information for clinical practice.

A previous report showed that a substantial number of patients positive for anti-ARS antibodies were classified as CADM [17]. Consistent with this finding, we observed less frequent muscle weakness/myalgia and lower serum CK and aldolase levels in the ARS group than in the non-ARS group. Furthermore, patients with CADM were more frequently found in the ARS group than in the non-ARS group (60.9% vs. 40.0%). In accordance with several previous studies showing that ILD often precedes PM/DM diagnosis in patients with anti-ARS antibodies [11, 32], the number of our patients with ILD-preceding type with no evidence of myositis at initial ILD diagnosis was larger in the ARS group than in the non-ARS group (21.7% vs. 8.0%). Collectively, the clinical features of anti-ARS-positive PM/DM-ILD patients were consistent with previous reports.

Radiologically, the NSIP pattern is the most common in PM/DM-ILD [5, 33]. Moreover, OP, diffuse alveolar damage (DAD), UIP, and mixed patterns with these features were also reported [5, 27, 33, 34]. Similar to PM/DM-ILD, the NSIP pattern is likely to be the most common in ASS-ILD, although various other HRCT patterns have also been reported [19–21, 32, 35, 36]. To date, there has been no report comparing HRCT patterns according to the current guidelines for IIPs [26, 27] between anti-ARS-positive and anti-ARS-negative PM/DM-ILD patients. In the present study, the NSIP pattern was significantly more frequent in the ARS group than in the non-ARS group, and HRCT patterns were more heterogeneous in the non-ARS group. Taken together, these results suggest that the presence of anti-ARS antibodies may affect HRCT pattern by influencing disease pathophysiology.

Pathologically, NSIP is also the most common pattern in PM/DM-ILD, followed by UIP and OP [4, 5, 8, 33, 37, 38]. DAD is often encountered in rapidly progressive ILD or autopsy cases [4–8, 33, 37, 38]. In ASS-ILD regardless of PM/DM diagnosis, NSIP is the primary histological pattern [32, 35, 39], but UIP, DAD, and OP are also reported [19–21]. In the present study, NSIP was the predominant pathological pattern in both ARS and non-ARS groups. UIP was pathologically diagnosed in only 8% of ARS and 14% of non-ARS cases, although mild fibroblastic foci and microscopic honeycombing were observed in some cases in this PM/DM-ILD cohort (Table 4). Pathological findings suggestive of underlying CTD [30], such as prominent plasmacytes or lymphoid aggregate with germinal center formation, were present in more than 50% of all PM/DM-ILD patients. There were no significant differences in pathological findings, although organization tended to be more frequent in the non-ARS group. Collectively, our data suggest that PM/DM-ILD patients primarily exhibit the NSIP pattern on lung histology, with no differences in pathological findings between anti-ARS-positive and anti-ARS-negative groups.

Several prognostic factors for PM/DM-ILD have been identified [4, 6–9, 13–15, 34]. Our previous study of 114 patients with PM/DM-ILD indicated that older age, acute/subacute form of ILD, lower FVC, and CADM diagnosis were associated with poor prognosis [9], whereas other reports identified the presence of anti-MDA-5 antibody and higher levels of serum ferritin as indices of poor prognoses [14, 15, 34]. Consistent with previous studies, we found that lower FVC percentage and acute/subacute form of ILD were indicators of poor prognosis in PM/DM-ILD patients. Furthermore, anti-ARS-positive PM/DM-ILD patients (ARS group) had better prognosis compared with anti-ARS-negative PM/DM-ILD patients (non-ARS group) as evidenced by Kaplan—Meier survival curves and Cox hazard analysis. Moreover, the response to the initial treatment was more favorable in the ARS group than in the non-ARS group. Thus, our data suggest that the presence of anti-ARS antibodies predicts better outcome as well as favorable response to the initial treatment in PM/DM-ILD patients.

It was suggested that the clinical features and prognosis may differ depending on the specific types of anti-ARS antibodies present in patients with ASS regardless of the CTD diagnosis [17, 18]. However, we could not find any clinical differences in our PM/DM-ILD cohort, possibly because of relatively smaller sample size (data not shown).

This study had several limitations. First, given its retrospective design and inclusion of ILD patients who visited a pulmonary division, it is subject to several possible biases. For instance, because the current authors’ institution is a regional referral center for ILD, referral or selection bias may have increased the number of patients with pulmonary manifestations. Second, because of the relatively small sample size, it was not possible to test whether the identified factors were independent risk factors by multivariate analysis in a Cox proportional hazards model. Third, it is possible that patients positive for anti-OJ antibody (or other anti-ARS antibodies not detected by ELISA) may have been included in the non-ARS group. However, the prevalence of these other anti-ARS antibodies was reported as rare (only 1%–5% of IIM patients) [16, 23]. Thus, it may not have significantly affected our results. Furthermore, MSAs other than anti-ARS antibodies were not measured in this study. Therefore, there is a possibility that some patients may have been positive for other MSAs, including anti-MDA-5 antibody, in our PM/DM-ILD cohort. Further study is needed to clarify this issue. Finally, the initial treatment regimen was not uniform. Most patients were treated with prednisolone (0.5–1.0 mg/kg per day) with/without immunosuppressants, such as cyclosporine, cyclophosphamide, or tacrolimus, for PM/DM-ILD. Given the rarity of PM/DM-ILD, it is unlikely that a larger, prospective, and randomized trial will be performed soon.

In conclusion, the present study demonstrated significant associations between the presence of anti-ARS antibodies and certain clinical features of PM/DM-ILD, such as female predominance and mild myositis. More importantly, positivity for anti-ARS antibodies predicted favorable response to treatment and longer survival. Therefore, measurement of anti-ARS antibodies provides important information for appropriate management of PM/DM-ILD patients.

## Supporting Information

S1 Protocol Definition of HRCT findings. (DOC)

S2 Protocol Definition of HRCT patterns. (DOC)
