# Supplementary material for: Prognostic Significance of Anti-Aminoacyl-tRNA Synthetase Antibodies in Polymyositis/Dermatomyositis-Associated Interstitial Lung Disease: A Retrospective Case Control Study
Source: PLoS One. 2015 Mar 19;10(3):e0120313. doi: 10.1371/journal.pone.0120313 (PMC4366175; doi:10.1371/journal.pone.0120313)
Supplement: S1 Protocol — (DOC) [file pone.0120313.s001.doc]

**S1 Protocol. Definition of HRCT findings.**

The HRCT findings, including ground-glass opacity (GGO), consolidation, reticular opacity, honeycombing, traction bronchiectasis, non-septal linear opacity/subpleural curvilinear line (SCLL), emphysema, and lower lobe volume loss were interpreted according to Fleischner’s criteria with slight modification [29]. GGO was defined as hazy increased attenuation of lung which did not obscure the underlying vessels. Consolidation was defined as homogeneous increase in pulmonary parenchymal attenuation that obscured the underlying vessels. Reticular opacity was considered as present when interlacing line shadows separated by a few millimeters were seen. Honeycombing was defined as the appearance of clustered cystic air spaces, typically of comparable diameters on the order of 3–10 mm but occasionally as large as 2.5 cm, in the subpleural regions, with well-defined walls. Traction bronchiectasis was defined as irregular bronchial dilatation within or around areas with parenchymal abnormality. Non-septal linear opacity was defined as elongated line of soft tissue attenuation distinct from interlobular septa and bronchovascular bundles. SCLL was defined as the finding that is a thin curvilinear line, 1-3 mm in thickness, lying less than 1cm from and parallel to the pleural surface. Emphysema was defined as focal region of low attenuation without visible walls. Lower lobe volume loss was defined as volume loss of bilateral lower lobes. Each CT finding was recorded as present or absent.
