# Supplementary material for: Prognostic Significance of Anti-Aminoacyl-tRNA Synthetase Antibodies in Polymyositis/Dermatomyositis-Associated Interstitial Lung Disease: A Retrospective Case Control Study
Source: PLoS One. 2015 Mar 19;10(3):e0120313. doi: 10.1371/journal.pone.0120313 (PMC4366175; doi:10.1371/journal.pone.0120313)
Supplement: S2 Protocol — (DOC) [file pone.0120313.s002.doc]

**S2 protocol. Definition of HRCT patterns.**

The HRCT pattern was classified as usual interstitial pneumonia (UIP) pattern, possible UIP pattern, or inconsistent with UIP pattern according to the guidelines for IPF with slight modification [26]. The cases interpreted as inconsistent with UIP pattern were further classified as nonspecific interstitial pneumonia (NSIP) pattern or organizing pneumonia (OP) pattern according to the guidelines for idiopathic interstitial pneumonias (IIPs) [25, 27]. Briefly, UIP pattern was characterized by subpleural and basal predominance, reticular abnormalities, honeycombing with/without traction bronchiectasis, and the absence of features listed as inconsistent with UIP pattern, including upper/mid-lung predominance or diffuse distribution, peribronchovascular predominance, extensive ground-glass abnormalities without reticular abnormalities (extent > reticular abnormalities with/without ground-glass abnormalities), profuse micronodules (at least > 10% of area in any of six lung zone), discrete cysts (multiple, bilateral, away from areas of honeycombing), diffuse mosaic attenuation/air-trapping (bilateral in three or more lobes) and consolidation in bronchopulmonary segment(s)/lobe(s). Possible UIP pattern was characterized by subpleural and basal predominance, reticular abnormalities, and the absence of features listed as inconsistent with UIP pattern. If the HRCT pattern did not meet the criteria, it was interpreted as inconsistent with UIP pattern. NSIP pattern was characterized by broad ground-glass attenuation more than reticulation without/with minimal honeycombing without prominent consolidation in diffuse or peribronchovascular distribution of predominant bilateral lower lobe. OP pattern was characterized by patchy consolidation in subpleural, peribronchial distribution, or band-like pattern, commonly associated with ground-glass opacity. Patterns that could not be classified as NSIP or OP were categorized as unclassifiable pattern. Disagreements regarding HRCT interpretation were resolved by a consensus between the two radiologists.
